# Integrated Management of a Large Radicular Cyst: A Case Report on Efficacy of Decompression and Conservative Approaches

**DOI:** 10.1002/ccr3.70070

**Published:** 2025-01-06

**Authors:** Ali Chamani, Siavash Moushekhian, Mina Zarei, Reza Shakiba, Mohammad Hossein Davarian

**Affiliations:** ^1^ Department of Endodontics Mashhad University of Medical Sciences Mashhad Iran; ^2^ Dental Faculty Mashhad University of Medical Sciences Mashhad Iran

**Keywords:** decompression, endodontic surgery, GTR, radicular cyst

## Abstract

The decompression technique can effectively reduce the size of large periapical lesions, minimize tissue damage, and enhance surgical outcomes. This conservative approach allows for better management of extensive lesions, potentially improving patient recovery and decreasing the need for more invasive procedures.

## 1 Introduction

Periapical cysts, primarily classified as radicular cysts, are the most prevalent odontogenic cystic lesions of inflammatory origin, accounting for 50% of jaw cystic incidences [1]. These cysts arise from the epithelial cell rests of malassez in the periodontal ligament, stimulated by inflammation due to pulpal necrosis in non‐vital teeth [2]. They can be categorized into true and pocket cysts based on the communication between the cyst cavity and the root canal; true cysts, which don't have this connection, have a lower healing chance after root canal treatment, with an incidence of 8%–13% [3]. Factors such as increased osmotic pressure within the cyst contribute to their enlargement [4]. Apical periodontitis is commonly associated with dental caries and trauma which mainly affects the anterior maxilla [5]. Cone beam computed tomography (CBCT) enhances preoperative diagnostic accuracy due to its superior specificity and precision [6]. Cystic lesions can exhibit varying growth patterns, including enlargement, stagnation, or regression [7]. particularly in large lesions in the upper frontal region cyst expansion may not only impact the periodontal ligament but also the alveolar bone and adjacent anatomical structures such as the mandibular nerve and maxillary sinuses, potentially affecting normal functions [8]. Additionally, extra complications such as dental dislocations, pathological fractures, and facial asymmetry may occur. Histopathological examination is essential for accurately diagnosing lesions as either granulomas or cysts [9].

Early diagnosis and treatment of cystic lesions are crucial. A non‐surgical endodontic approach is preferred, but surgical management may be necessary in cases where routine endodontic treatment fails or is not possible [10]. True periapical cysts are mainly considered self‐sustaining, often remaining despite removing original microbiological stimuli in the root canal, necessitating surgical intervention [3]. Post‐root canal treatment follow‐up is essential before deciding on any surgical procedures. The main factors influencing the decision for surgical intervention include lesion size, tooth location, and proximity to vital anatomical structures [11]. Since Surgical techniques can cause complications such as postoperative pain and damage to adjacent structures, it has been recommended to use them only when less invasive methods prove ineffective [12]. Decompression, first described by Dr. Carl Partsch, is a conservative management option for large cystic lesions, causing size reduction before enucleation [13, 14]. Decompression alleviates internal hydrostatic pressure of the cyst and assists peripheral bone regeneration, it has also shown considerable effectiveness, reducing cystic areas by an average of 79.3%, making it a favorable approach in managing odontogenic cysts [15].

This case report is reported for several reasons. First, it demonstrates the successful use of a combined treatment approach—decompression followed by surgical excision—for a large radicular cyst, highlighting the efficacy of less invasive approaches in the management of complex cases. Second, this case highlights the innovative use of various clinical tools to aid in decompression, leading to positive outcomes, demonstrating how innovative strategies can lead to positive outcomes. Finally, this report contributes to the ongoing debate about the role of guided tissue regeneration (GTR) in the treatment of extensive lesions and suggests that further research could enhance our understanding of its potential benefits.

## 2 Case Examination

The patient is a 34‐year‐old male with a dental history of childhood trauma to maxillary incisors. Three years before this evaluation, endodontic treatment was performed on the affected tooth, the maxillary left central (upper left 1); however, the problem persisted, and the tooth exhibited a mobility grade two. Reports of tooth sensitivity tests are present in Table 1. The patient's chief complaints included dull pain and a new diastema. General health status was reported by the patient as normal and in his last medical checkup. No issues were detected in his test results. the presence of a new diastema reported by the patient hinted at teeth movement.

**TABLE 1:** Report of tooth sensitivity assessments.

Tooth	ESP	Cold	Heat	Percussion	Palpation	Mobility
UR1	0	−	−	+	+	1
UL1	0	−	−	+	+	2
UL2	0	−	−	+	−	1
UL3	6	−	−	−	−	0
UL4	6	WNL	WNL	−	−	0

Abbreviation: WNL, within the normal limit.

## 3 Methods

### 3.1 Investigation

**FIGURE 1:**
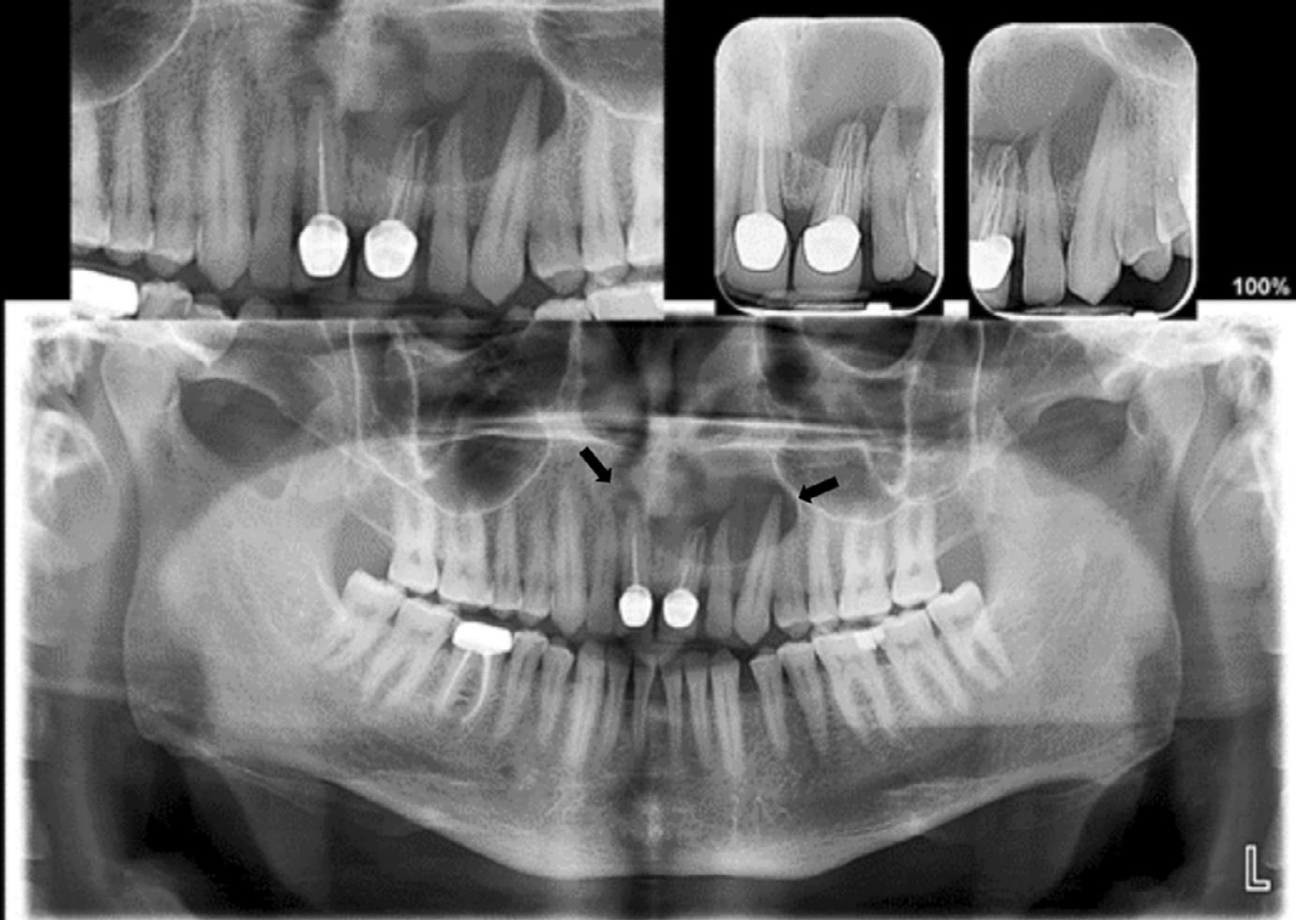
Initial OPG revealing a large radiolucent lesion in the upper left quadrant. Arrows pointing at the large radicular cyst and the radiolucency at UR1.

Panoramic radiographic imaging indicated that the upper left central incisor of the maxilla, which had been previously treated, exhibited poor endodontic treatment (RCT) and a large periapical lesion that extended from slightly beyond the midline to encompass the mesial side of the buccal root of the left first premolar (Figure 1).

**FIGURE 2:**
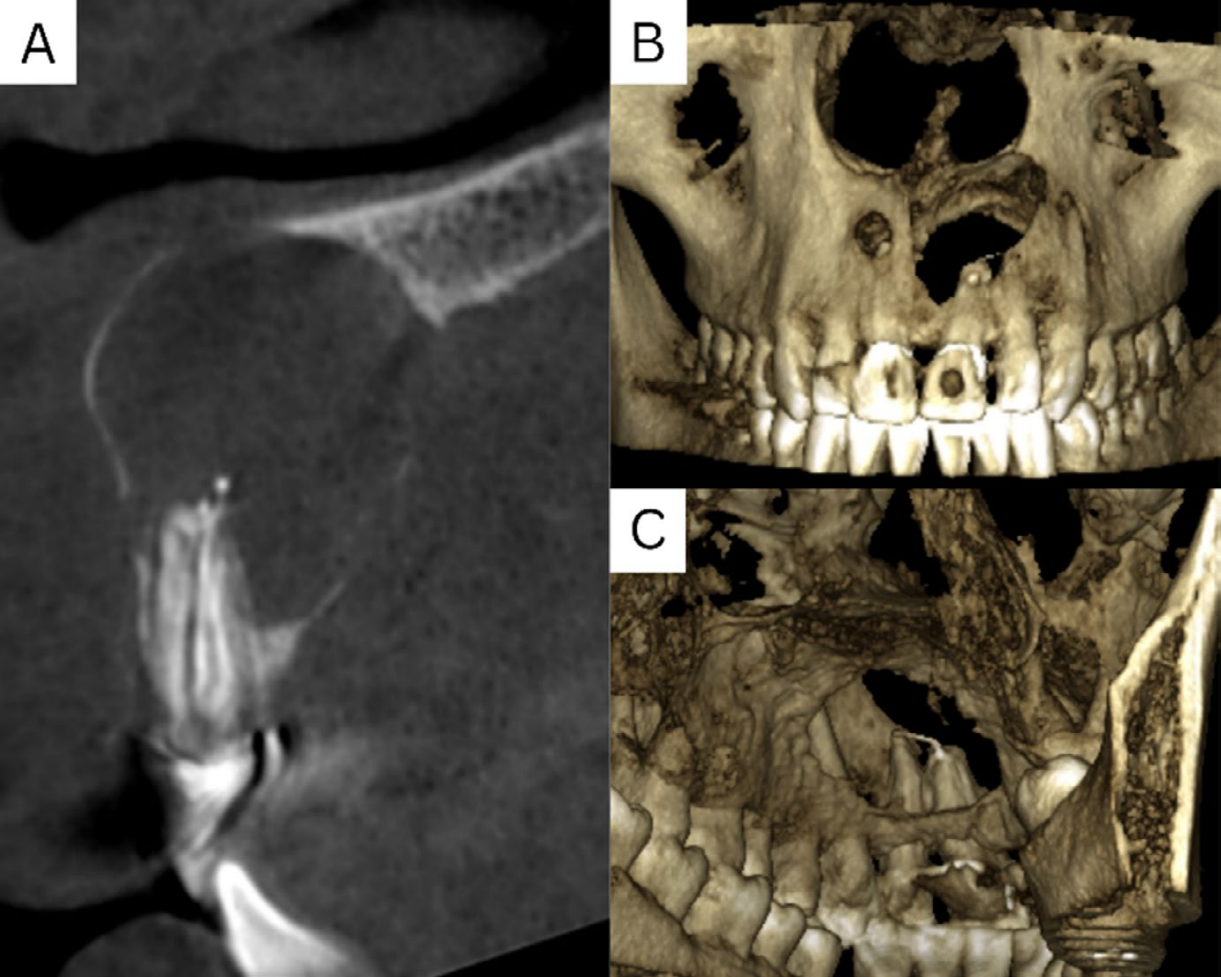
(A) CBCT sagittal section showing a large radiolucency with thinning of the labial and palatal bone cortex. (B) 3D reconstruction reveals labial perforation and a separate lesion at the apex of the upper right central incisor that requires examination. (C) Alternate 3D angle highlighting the palatal perforation and inadequate obturation extending beyond the apical foramen.

A Cone Beam Computed Tomography (CBCT) was requested, and a significant destructive cyst was apparent. Notably, both the labial and palatal bone cortex had become extremely thin, with observed perforations in both the labial and palatal cortical plates (Figure 2).

### 3.2 Differential Diagnosis

A radiolucent lesion in the maxilla with well‐defined borders and expansion and extension with no root resorption present in relation to UL1. The differential diagnosis included (1) radicular cyst, (2) odontogenic keratocyst (OKC), and (3) ameloblastoma.

### 3.3 Treatment

**FIGURE 3:**
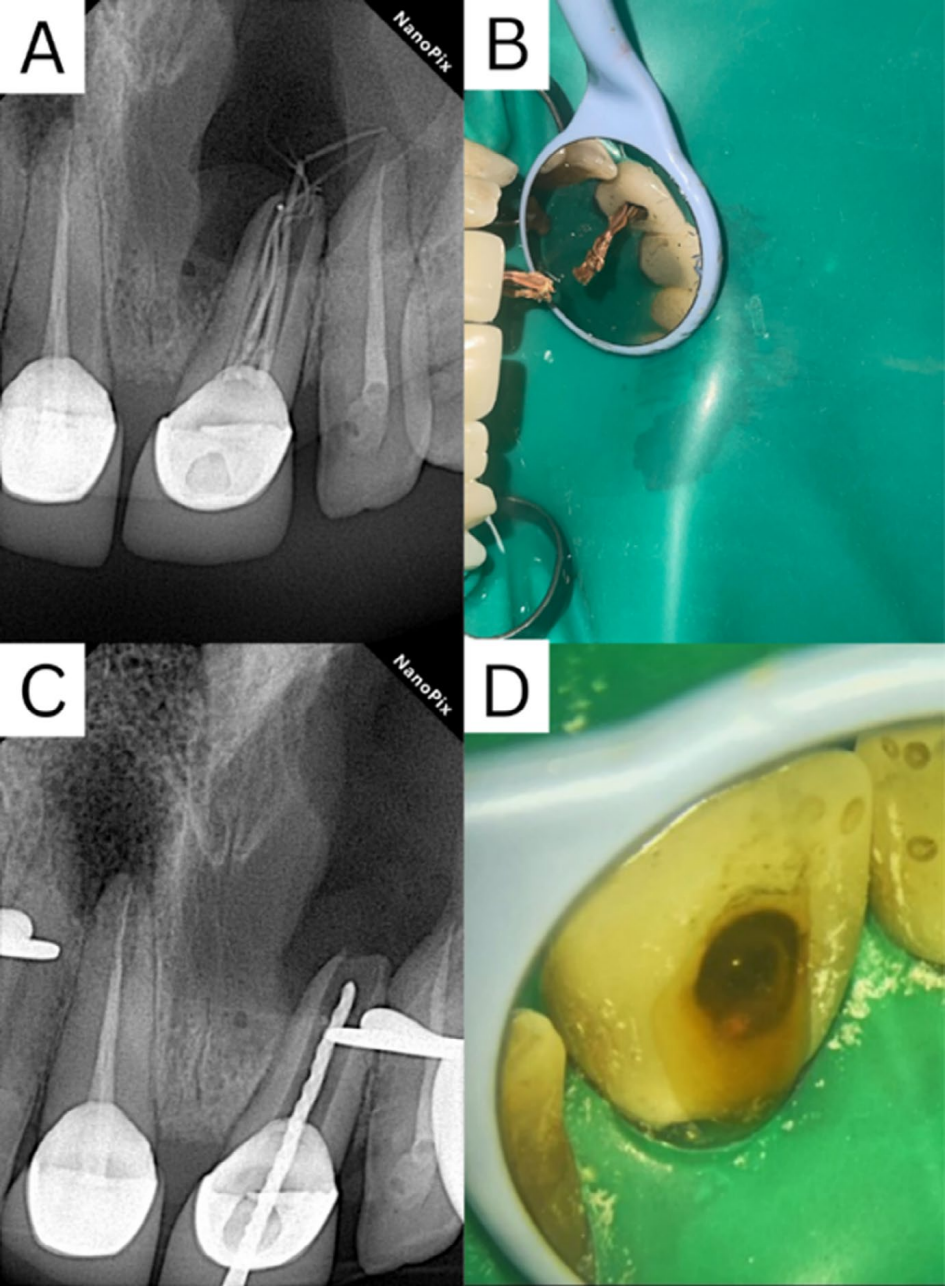
(A) Initial periapical radiograph showing insufficient obturation and the presence of a lesion at the apex of the upper right central incisor. (B) Removed gutta‐percha (C) Periapical radiograph showing the complete removal of the previous fillings. (D) Clinical picture showing persistent pus flow from the access site, indicating ongoing infection.

Initially, a non‐surgical treatment approach was executed. Following the removal of the previous root fillings, significant purulent secretion made further obturation impossible, so calcium hydroxide was placed in the canal (Figure 3).

Two weeks later, upon follow‐up, the patient returned with ongoing discharge upon removal of the temporary filling, other methods of orthograde fillings were tested, but MTA angelus (Angelus Industry, Londrina, Brazil) application in apex was not applicable and it nearly was pushed into the cyst, as all attempts failed and the persistent flow of puss indicating that the orthograde approach was insufficient for resolving the condition. Therefore, the treatment plan was revised to include decompression and marsupialization, considering the size of the lesion and the potential for tissue deformation if the surgical intervention happened immediately.

Given the failure of the orthograde approach to control the purulent discharge and the size of the lesion, it was deemed necessary to proceed with a retrograde approach that included decompression.

**FIGURE 4:**
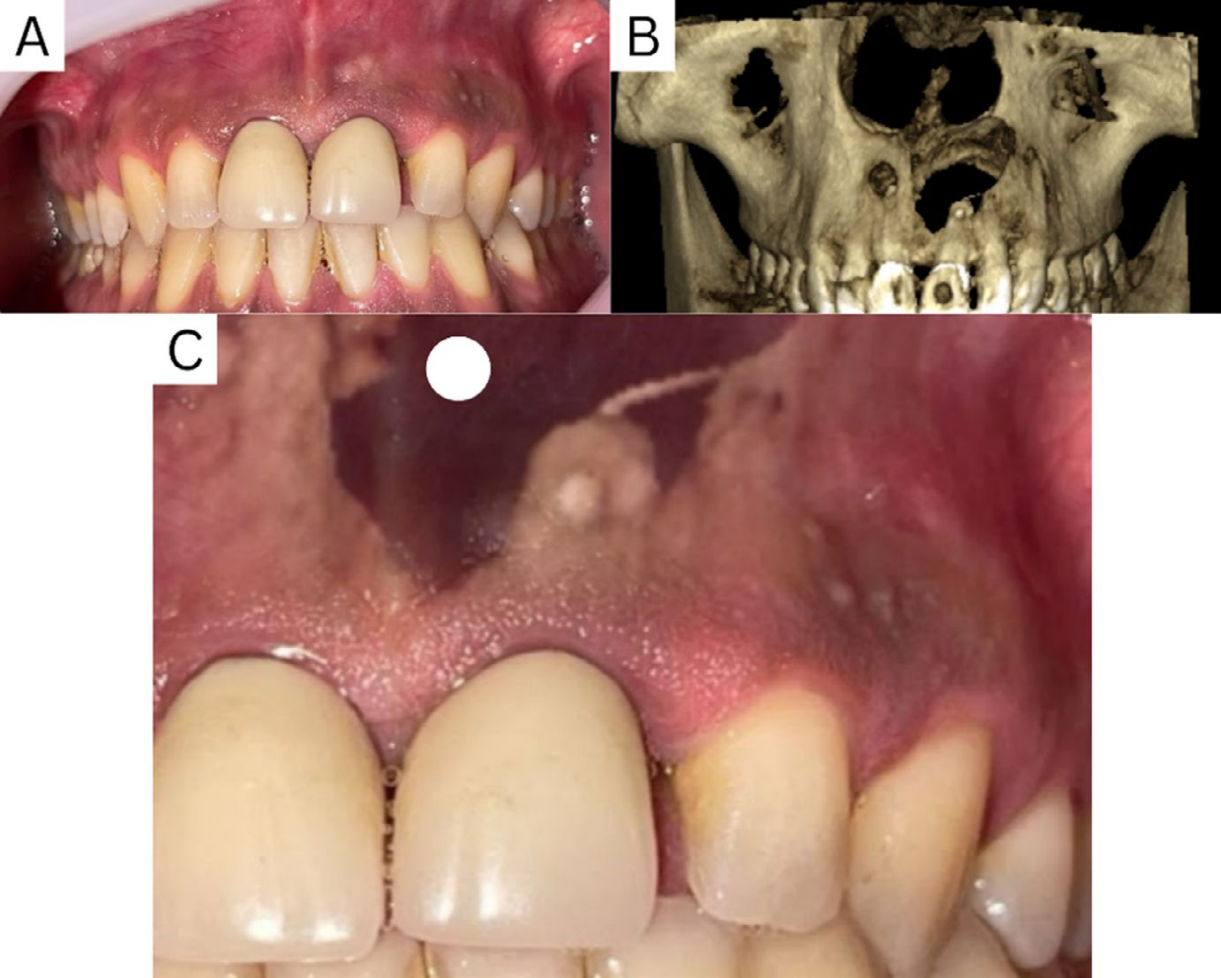
(A) Intraoral photograph of the patient. (B) CBCT 3D image reconstruction from the same angle. (C) Merged images allowing for precise determination of the extent of the lesion and optimal incision site.

The patient provided informed consent for both the treatment and surgical procedure. An infiltration injection of lidocaine 2% epinephrine 1/100,000 (Xylopen, Tehran, Iran) at UL1, the full carpool was injected. The site of the incision was determined by integrating the patient's CBCT with an intraoral photograph, finding the optimal location for the incision (Figure 4). A 1 mm vertical incision was performed in the soft tissue and upon incision no exodus exit was noticed since the cyst wall wasn't penetrated.

**FIGURE 5:**
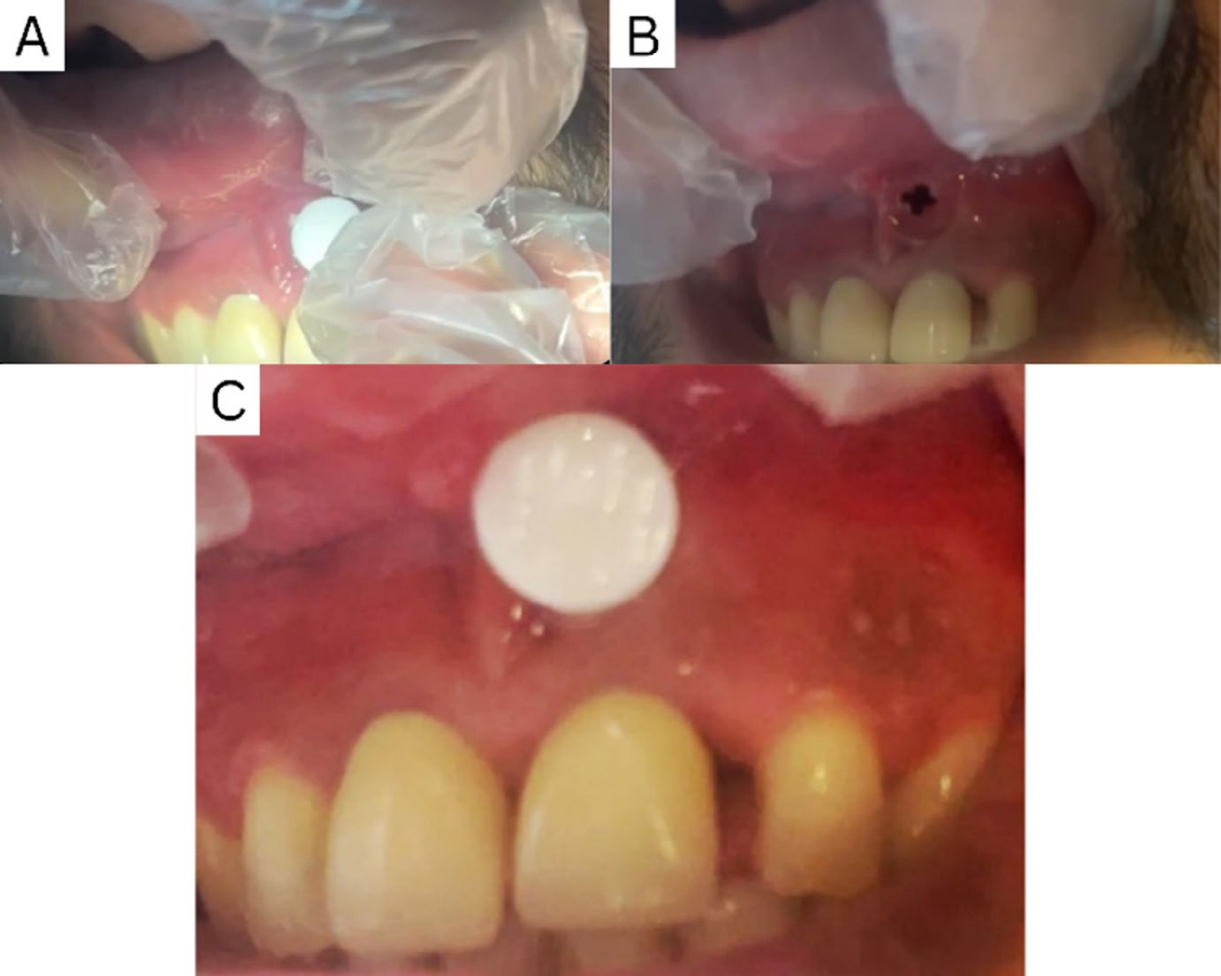
(A) The Customized Plunger of the syringe was inserted in the lesion at a predetermined length. (B) The soft tissue conformed to the shape of the syringe after insertion. (C) The syringe in its place to keep the lumen open.

The desired drain needs qualities such as biocompatibility, adequate size to prevent clogging, stability in the oral biome, ease of maintenance for the patient, patient comfort, monitoring capability, etc. An insulin syringe was cut to the appropriate length corresponding to the center of the radiolucency, measured at 19.80 mm via CBCT from the labial cortical bone to the center of the lesion. The syringe was put through a disinfection procedure that included irrigation with betadine, followed by isopropyl alcohol, and then rinsed with sterile normal saline, respectively. The drain was carefully inserted. After the drain penetrated the cyst wall, exodus started to be drained. After the surgery, the patient was prescribed to rinse his mouth with chlorohexidine 0.2% for 10 days. The patient was instructed to rinse the lumen with saline three times daily and was educated on the proper technique for reinserting and removing the drain (Figure 5). The patient was asked for any complaints about the usage or esthetic use of the drain. Since the insertion plan was not in the smile line no esthetic complaints were given, the only complaint was a bad taste of mouth especially after sleep which was from continuous drainage of the exodus.

**FIGURE 6:**
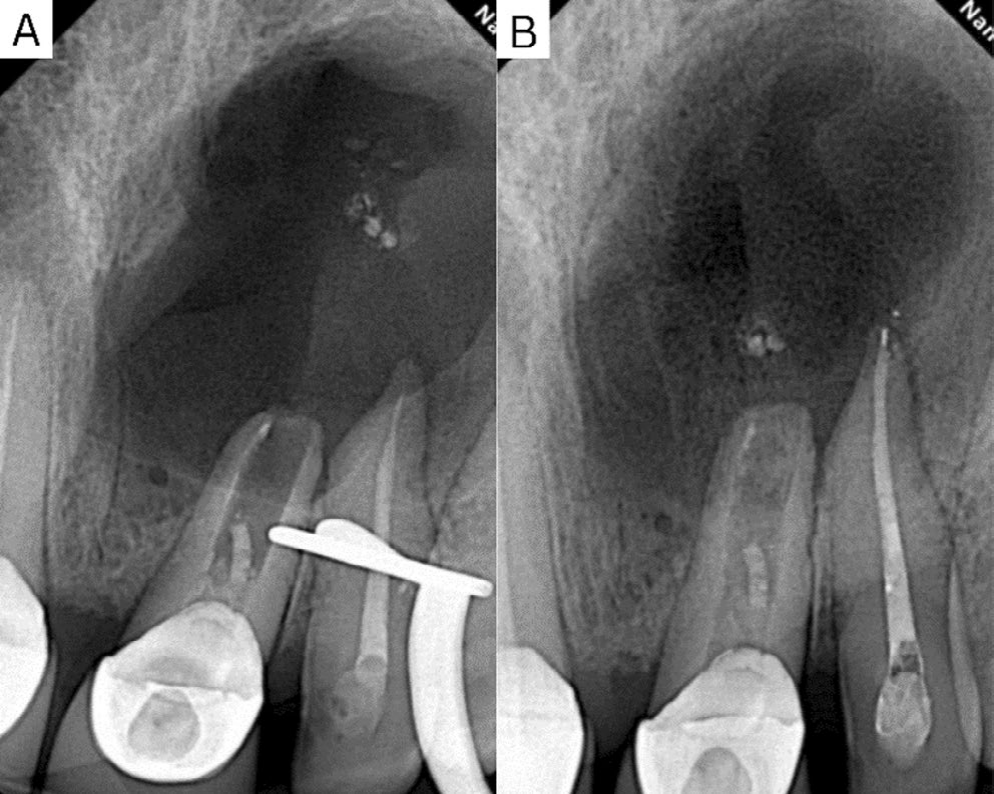
(A) Immediate periapical radiograph after decompression. (B) Follow‐up radiograph after 3 months demonstrating bone healing following pressure relief from the internal lesion. The upper left lateral was also retreated in the meantime due to inadequate past obturation.

Recall periapical radiographs taken three months post‐decompression demonstrated a progressive reduction of lesion size and an increase in the density of the regenerated trabecular bone (Figure 6).

**FIGURE 7:**
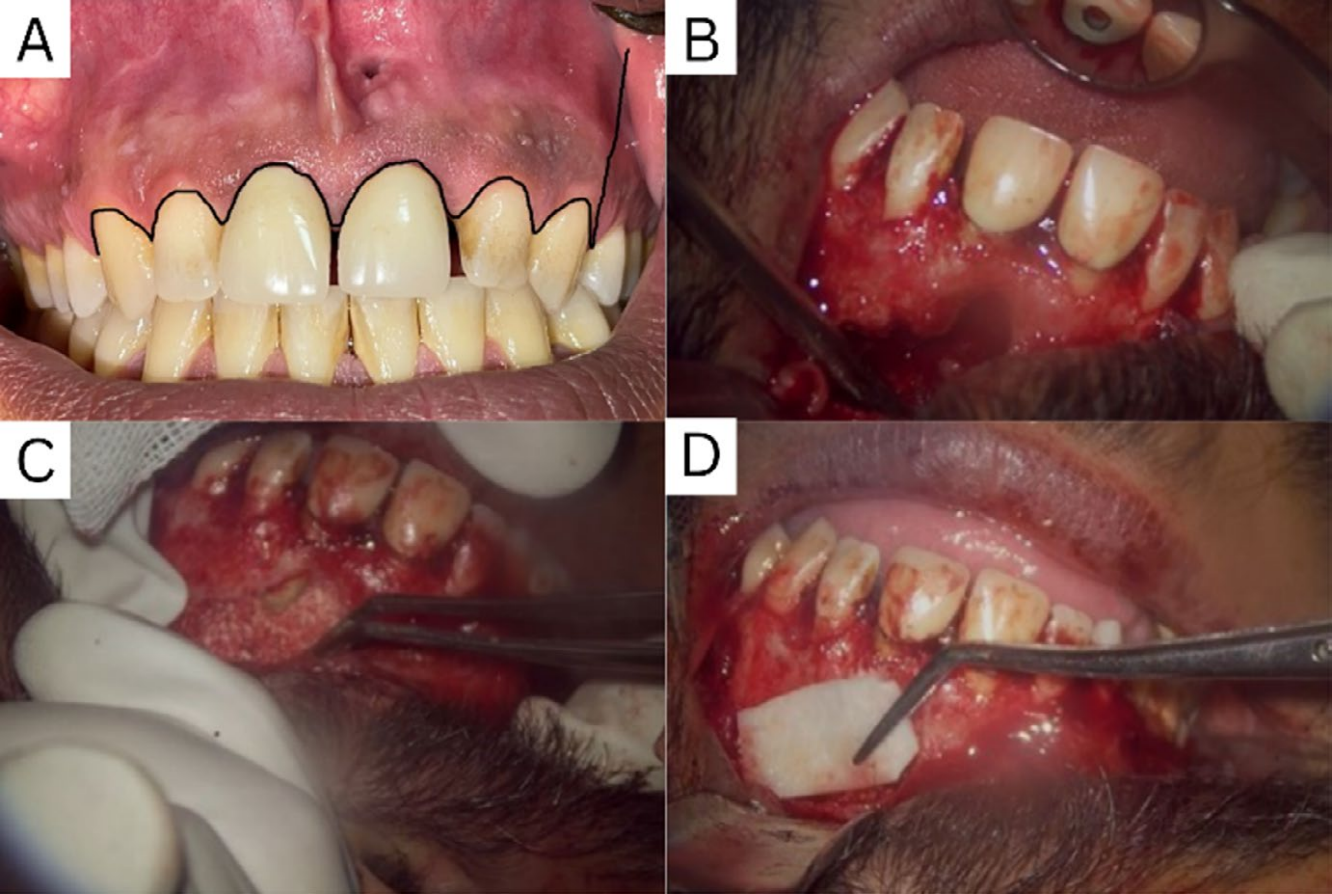
(A) Flap design for surgical access. (B) The cyst was excised, and the site was thoroughly irrigated and curetted to ensure complete cyst wall removal. (C) The cavity was filled with allograft‐derived matrix Cenobone (Iran). (D) The labial perforation was covered with Cenomembrane (Iran).

Once the lesion had reached the desired size, two bilateral infraorbital injections of Lidocaine 2% epinephrine 1/100,000 (Xylopen, Tehran, Iran) were injected. A full mucoperiosteal triangular flap was made, the flap design extending from the right to the left canine, with the releasing incision at the distobuccal angle of the left canine. The cyst was excised, and the surgical site was thoroughly irrigated. The root canal was subsequently filled with MTA angelus (Angelus Industry, Londrina, Brazil). Due to the chance of deformity because of the lesion size, the cavity was additionally filled with an allograft‐derived matrix (Cenobone, Kish, Iran), and the labial perforation was covered with a membrane (Cenomembrane, Kish, Iran). The flap was closed using multiple single sutures. Additionally, the upper right central incisor (UR1) and upper left second incisor (UL2) underwent successful root canal treatment (RCT) as part of the comprehensive management plan (Figure 7).

**FIGURE 8:**
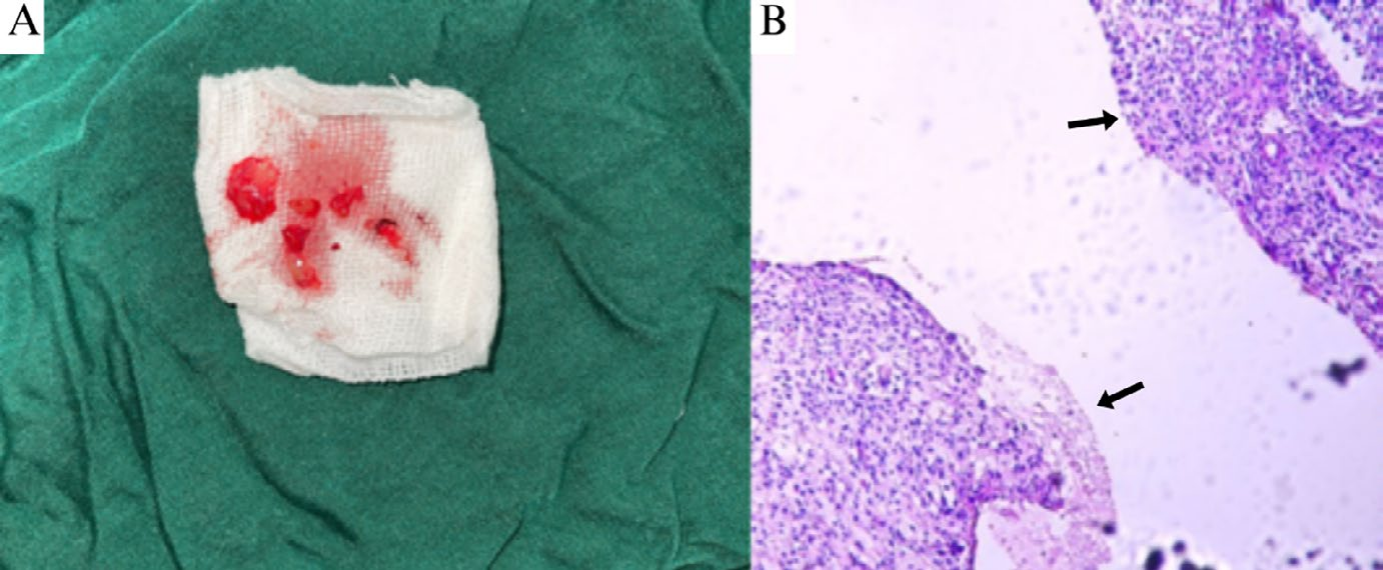
(A) Excised cyst wall removed from the lesion. (B) Pathological view of the cyst in 100× showing a typical histopathological view of an infectious odontogenic cyst with one layer of nonkeratinized stratified squamous epithelium and inflammatory cells infiltration. Some part of the cyst contained secretion of keratin.

Pathological examination of the excised cyst showed a significant infiltration of lymphoplasmic cells along with parts of blood vessels and collagen fibers related to the cyst wall. The cyst wall showed partial coverage by hyperplastic squamous epithelium with obvious exocytosis in the epithelium. The pathologist subsequently confirmed the diagnosis of an infectious odontogenic cyst (Figure 8).

## 4 Conclusion

### 4.1 Outcome and Follow‐Up

**FIGURE 9:**
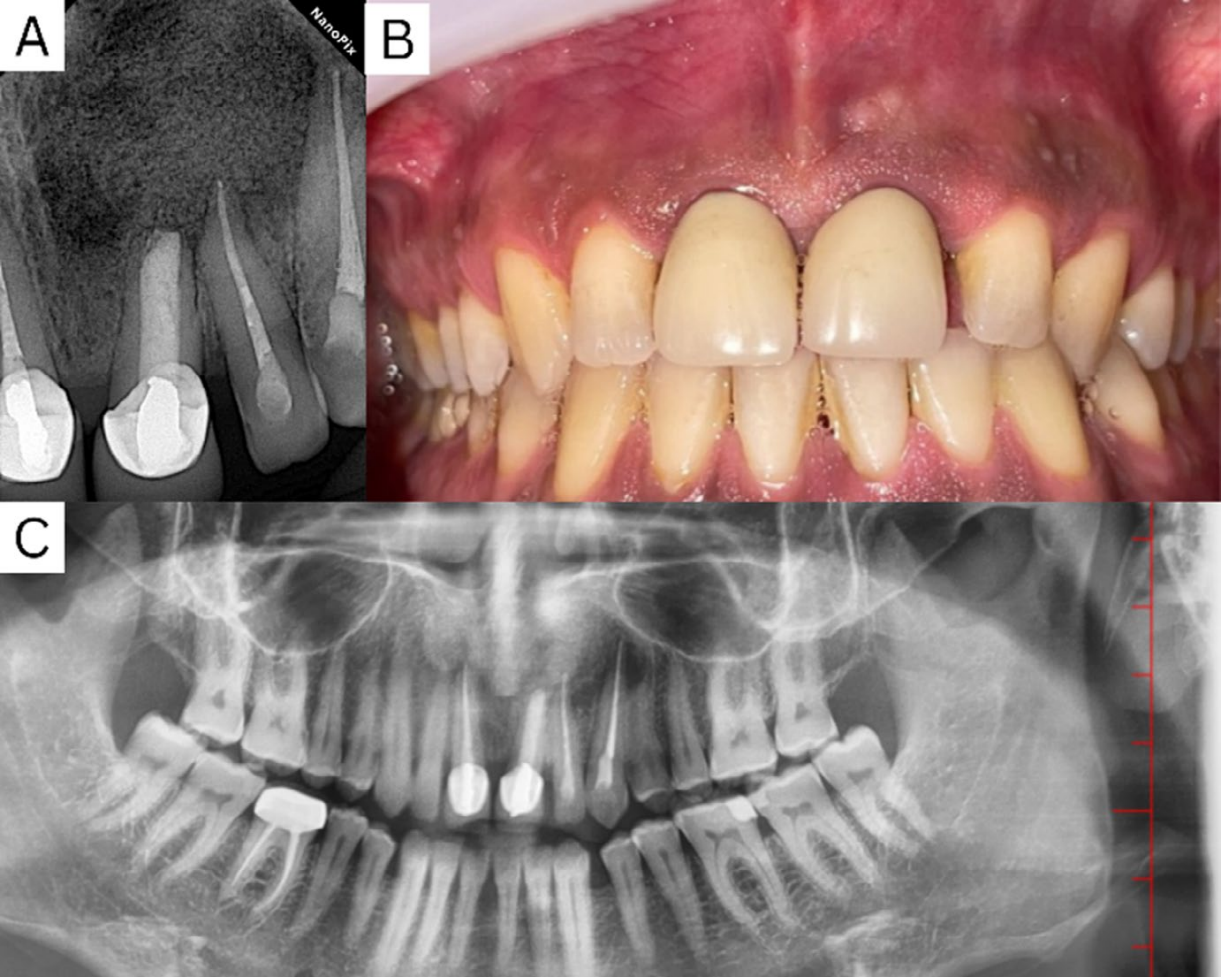
(A) Periapical radiograph demonstrating significant healing and osseointegration. (B) Intraoral view illustrating the absence of periodontal inflammation, with complete flap and lumen healing. (C) Panoramic radiograph showing overall healing, with newly treated teeth exhibiting healthy periapical structures.

After six months, complete healing of the lesion was observed, and the patient had no further complaints or complications (Figure 9). The UL4 showed normal sensitivity and healthy pulp diagnosis.

## 5 Discussion

Root canal therapy is often the first line of treatment for radicular cysts. However, the persistence of large periapical lesions is common in the case of non‐surgical interventions. This persistence can occur due to persistent intracanal or extra‐radicular infections and stimuli that slow down healing [16]. Multiple studies have highlighted the limitations of clinical and radiographic examinations in accurately diagnosing periapical lesions. Histopathological analysis remains the gold standard for definitive diagnosis. Advanced imaging techniques, such as CBCT has high accuracy (86%) in differentiating periapical cysts and granulomas, potentially reducing the need for invasive biopsy procedures [17, 18].

In cases where despite endodontic treatment using intracanal medications such as calcium hydroxide persistent discharge continues [19], alternative methods may be needed. Decompression is a viable alternative to more aggressive techniques for managing large cystic oral lesions [20], and can provide a solution for large cystic lesions. This approach, which involves making a hole in the cyst wall to facilitate drainage, aims to reduce intra‐cystic pressure and improve the healing of the surrounding tissues. The primary indications of decompression include large cysts greater than 5 cm where enucleation could compromise surrounding structures or where the patient has high surgical risks [21] also patients with complications that require a less invasive approach in their surgical treatment benefit from decompression compared to enucleation [22]. Bone regeneration is one of the main benefits of decompression, slow bone regeneration helps in maintaining the surrounding structures, and the defects that are common with enucleation of large lesions are avoided [23].

While decompression has several advantages, there are also contraindications to consider, decompression in small odontogenic cysts not only didn't show any significant benefit but also prolonged the treatment duration [22]. If the cyst is companied by an active infection, immediate enucleation may be put to address the infection more efficiently, if the lesion is suspected to have neoplastic tendencies and is not completely odontogenic, enucleation for complete removal, and immediate histopathological evaluation is necessary [21].

Decompression, especially compared to techniques such as the modified Partesh method (Partesh II), is aimed at minimizing trauma to adjacent anatomic structures, including the nasal floor or mandibular nerve canal [24]. The Partsch II procedure, also known as enucleation with primary closure, is a surgical technique that' is characterized by the complete removal of the cystic lesion along with its lining to ensure complete cyst removal and reduce the risk of recurrence and when there is a concern about residual cysts and a potential malignancy. The procedure is followed by early closure of the surgical site over the effect of fewer post‐operative complications and faster healing [25, 26]. In the presented case, a primary enucleation not only endangers the nasal floor but could also affect the adjacent UL4 tooth, and pulpal vitality is compromised. Furthermore, with decompression, a total enucleation was possible, so decompression was put in order.

Decompression effectively reduces the size of jaw cystic lesions, especially when associated with longer treatment duration, younger patient age, and posterior maxilla location. Decompression has been reported to reduce cyst size, lead to faster bone regeneration, and make restoration with dental implants in the location of the lesion in the mandible possible [23, 27]. However, a complete endodontic treatment must be performed before decompression procedures to distinguish a true cyst from a pseudocyst [7]. This multi‐session method requires patient cooperation and regular follow‐ups to monitor improvement and prevent complications.

In cases where the lesion is significantly large, simply removing the cyst may cause tissue deformity after cyst removal due to the large volume of destruction. The use of GTR techniques can be particularly useful in the management of extensive periapical lesions. GTR protocols help isolate the lesion, facilitate repopulation by periodontal ligament and bone cells, and inhibit the proliferation of connective and epithelial cells by accelerating their division cycles [28]. GTR in periodontal and periapical surgery aims to prevent epithelial migration and promote connective tissue attachment to the root surface. Although complete regeneration of periapical tissues has been observed even in the absence of GTR protocols [29, 30], GTR incorporation is often considered essential in non‐containing defects associated with larger lesions. Despite some authors advocating the use of GTR as an adjunctive therapy for the surgical treatment of root‐periodontal lesions, the existing literature remains inconclusive regarding the definitive role of GTR in such interventions [31]. However, in the presented case, the use of GTR methods brought very interesting results.

Management of radicular cysts requires a comprehensive understanding of both conservative and surgical treatment methods. Decompression techniques are effective in reducing the size of large periapical lesions, thereby minimizing tissue damage and improving surgical outcomes. This conservative approach not only allows for better management of extensive lesions but also has the potential to improve patient recovery and reduce the need for more invasive procedures.

When conventional endodontic treatments have failed to resolve the problem, decompression can be especially helpful, providing relief and healing for larger lesions. In addition, the use of GTR may provide improved results for extensive defects. However, there is a clear need for further research to elucidate the most effective treatment protocols. Together, these strategies emphasize the importance of a balanced approach to the management of radicular cysts and highlight the balance between conservative measures and surgical interventions in optimizing patient outcomes.

## Author Contributions

**Ali Chamani:** conceptualization, investigation, methodology, project administration, supervision, visualization. **Siavash Moushekhian:** conceptualization, supervision, visualization. **Mina Zarei:** writing – original draft, writing – review and editing. **Reza Shakiba:** writing – original draft, writing – review and editing. **Mohammad Hossein Davarian:** data curation, investigation, resources, writing – original draft, writing – review and editing.

## Consent

Written informed consents were obtained from the patient to publish this case following the journal's patient consent policy.

## Conflicts of Interest

The authors deny any conflicts of interest.

## Data Availability

The data that support the findings of this study are available from the corresponding author upon reasonable request.

## References

[1] S. Kaur , H. Gupta , and H. Singh , “Radicular Cyst Classic Presentation: A Case Report and Review of Clinical, Radiological and Histopathological Features,” Journal of Dental and Medical Sciences 16, no. 4 (2017): 103–106.

[2] K. Karamifar , A. Tondari , and M. A. Saghiri , “Endodontic Periapical Lesion: An Overview on the Etiology, Diagnosis and Current Treatment Modalities,” European Endodontic Journal 5, no. 2 (2020): 54–67. 32766513 10.14744/eej.2020.42714 PMC7398993

[3] D. Ricucci , S. Loghin , J. F. Siqueira, Jr. , and R. A. Abdelsayed , “Prevalence of Ciliated Epithelium in Apical Periodontitis Lesions,” Journal of Endodontics 40, no. 4 (2014): 476–483. 24666895 10.1016/j.joen.2013.12.014

[4] N. Y. Yang , Y. Zhou , H. Y. Zhao , X. Y. Liu , Z. Sun , and J. J. Shang , “Increased Interleukin 1α and Interleukin 1β Expression Is Involved in the Progression of Periapical Lesions in Primary Teeth,” BMC Oral Health 18, no. 1 (2018): 124. 30012121 10.1186/s12903-018-0586-3 PMC6048863

[5] C. Tibúrcio‐Machado , C. Michelon , F. Zanatta , M. S. Gomes , J. A. Marin , and C. A. Bier , “The Global Prevalence of Apical Periodontitis: A Systematic Review and Meta‐Analysis,” International Endodontic Journal 54, no. 5 (2021): 712–735. 33378579 10.1111/iej.13467

[6] B. Pitcher , A. Alaqla , M. Noujeim , J. A. Wealleans , G. Kotsakis , and V. Chrepa , “Binary Decision Trees for Preoperative Periapical Cyst Screening Using Cone‐Beam Computed Tomography,” Journal of Endodontics 43, no. 3 (2017): 383–388. 28231977 10.1016/j.joen.2016.10.046

[7] F. C. Tian , B. E. Bergeron , S. Kalathingal , et al., “Management of Large Radicular Lesions Using Decompression: A Case Series and Review of the Literature,” Journal of Endodontics 45, no. 5 (2019): 651–659. 30833094 10.1016/j.joen.2018.12.014

[8] L. Oliveros‐Lopez , A. Fernandez‐Olavarria , D. Torres‐Lagares , et al., “Reduction Rate by Decompression as a Treatment of Odontogenic Cysts,” Medicina Oral, Patología Oral y Cirugía Bucal 22, no. 5 (2017): e643–e650. 28809378 10.4317/medoral.21916 PMC5694189

[9] L. M. Lin , D. Ricucci , and B. Kahler , “Radicular Cysts Review,” JSM Dental Surgery 2, no. 2 (2017): 1017.

[10] R. M. Talpos‐Niculescu , M. Popa , L. C. Rusu , M. O. Pricop , L. M. Nica , and S. Talpos‐Niculescu , “Conservative Approach in the Management of Large Periapical Cyst‐Like Lesions. A Report of Two Cases,” Medicina (Kaunas) 57, no. 5 (2021): 497. 34068934 10.3390/medicina57050497 PMC8156608

[11] K. Sheth , S. Kapoor , and S. Daveshwar , “Comparison of Cone‐Beam Computed Tomography and Periapical Radiography to Determine the Proximity of Periapical Lesions to Anatomical Structures in Premaxillary Area Prior to Surgical Endodontics: A Clinical Study,” International Journal of Clinical Pediatric Dentistry 13, no. 4 (2020): 322–326. 33149402 10.5005/jp-journals-10005-1783 PMC7586484

[12] M. Del Fabbro , S. Corbella , P. Sequeira‐Byron , et al., “Endodontic Procedures for Retreatment of Periapical Lesions,” Cochrane Database of Systematic Reviews 10 (2016): CD005511. 27759881 10.1002/14651858.CD005511.pub3 PMC6461161

[13] J. Castro‐Núñez , “Decompression of Odontogenic Cystic Lesions: Past, Present, and Future,” Journal of Oral and Maxillofacial Surgery 74, no. 1 (2016): 104. e1–104.e9. 10.1016/j.joms.2015.09.004 26428611

[14] R. Wakolbinger and J. Beck‐Mannagetta , “Long‐Term Results After Treatment of Extensive Odontogenic Cysts of the Jaws: A Review,” Clinical Oral Investigations 20, no. 1 (2016): 15–22. 26250795 10.1007/s00784-015-1552-y

[15] L. Oliveros‐Lopez , A. Fernandez‐Olavarria , D. Torres‐Lagares , et al., “Reduction Rate by Decompression as a Treatment of Odontogenic Cysts,” Medicina Oral, Patologia Oral y Cirugia Bucal 22, no. 5 (2017): e643. 28809378 10.4317/medoral.21916 PMC5694189

[16] L. M. Lin , D. Ricucci , J. Lin , and P. A. Rosenberg , “Nonsurgical Root Canal Therapy of Large Cyst‐Like Inflammatory Periapical Lesions and Inflammatory Apical Cysts,” Journal of Endodontics 35, no. 5 (2009): 607–615. 19410070 10.1016/j.joen.2009.02.012

[17] O. Alotaibi , S. Alswayyed , R. Alshagroud , and M. AlSheddi , “Evaluation of Concordance Between Clinical and Histopathological Diagnoses in Periapical Lesions of Endodontic Origin,” Journal of Dental Sciences 15, no. 2 (2020): 132–135. 32595891 10.1016/j.jds.2020.01.007 PMC7305430

[18] S. A. Soma , M. M. Akhtar , S. Begum , et al., “Evaluation of Cone‐Beam Computed Tomography to Differentiate Odontogenic Periapical Cysts and Granulomas,” Journal of National Institute of Neurosciences Bangladesh 8, no. 2 (2022): 171–174.

[19] B. Athanassiadis , P. V. Abbott , and L. J. Walsh , “The Use of Calcium Hydroxide, Antibiotics and Biocides as Antimicrobial Medicaments in Endodontics,” Australian Dental Journal 52, no. 1 Suppl (2007): S64–S82. 17546863 10.1111/j.1834-7819.2007.tb00527.x

[20] S. Sammut , A. Morrison , V. Lopes , and N. Malden , “Decompression of Large Cystic Lesions of the Jaw: A Case Series,” Oral Surgery 5, no. 1 (2012): 13–17.

[21] S. Marin , B. Kirnbauer , P. Rugani , A. Mellacher , M. Payer , and N. Jakse , “The Effectiveness of Decompression as Initial Treatment for Jaw Cysts: A 10‐Year Retrospective Study,” Medicina Oral, Patología Oral y Cirugía Bucal 24, no. 1 (2019): e47. 30573706 10.4317/medoral.22526 PMC6344015

[22] D. Shi , H. Dong , B. Chen , Z. Zhu , and T. Zhang , “Decompression‐First or Direct Enucleation: The Choice of Treatment for Medium‐Sized Odontogenic Jaw Cysts,” Journal of Stomatology, Oral and Maxillofacial Surgery 125 (2024): 101892. 38670344 10.1016/j.jormas.2024.101892

[23] M. AboulHosn , Z. Noujeim , N. Nader , and A. Berberi , “Decompression and Enucleation of a Mandibular Radicular Cyst, Followed by Bone Regeneration and Implant‐Supported Dental Restoration,” Case Reports in Dentistry 2019, no. 1 (2019): 9584235. 30729045 10.1155/2019/9584235 PMC6343149

[24] F. Riachi and C. Tabarani , “Effective Management of Large Radicular Cysts Using Surgical Enucleation vs. Marsupialization–Two Cases Report,” International Arab Journal of Dentistry (IAJD) 1, no. 1 (2010): 44–51.

[25] K. Sokler , S. Sandev , and J. Grgurević , “Surgical Treatment of Large Mandibular Cysts,” Acta Stomatologica Croatica 35, no. 2 (2001): 245–251.

[26] W. A. Abdullah , “Surgical Treatment of Keratocystic Odontogenic Tumour: A Review Article,” Saudi Dental Journal 23, no. 2 (2011): 61–65. 24151416 10.1016/j.sdentj.2011.01.002 PMC3770236

[27] Y.‐J. Kwon , K.‐S. Ko , B.‐K. So , et al., “Effect of Decompression on Jaw Cystic Lesions Based on Three‐Dimensional Volumetric Analysis,” Medicina 56, no. 11 (2020): 602. 33182601 10.3390/medicina56110602 PMC7696604

[28] L. Nibali , V. P. Koidou , M. Nieri , L. Barbato , U. Pagliaro , and F. Cairo , “Regenerative Surgery Versus Access Flap for the Treatment of Intra‐Bony Periodontal Defects: A Systematic Review and Meta‐Analysis,” Journal of Clinical Periodontology 47 (2020): 320–351. 31860134 10.1111/jcpe.13237

[29] I. Tsesis , V. Faivishevsky , A. Kfir , and E. Rosen , “Outcome of Surgical Endodontic Treatment Performed by a Modern Technique: A Meta‐Analysis of Literature,” Journal of Endodontics 35, no. 11 (2009): 1505–1511. 19840638 10.1016/j.joen.2009.07.025

[30] S. V. Wagle , A. A. Agrawal , D. Bardoliwala , and C. Patil , “Guided Tissue Regeneration,” Journal of Oral Research and Review 13, no. 1 (2021): 46–49.

[31] E. Rosen , I. Tsesis , E. Kavalerchik , et al., “Effect of Guided Tissue Regeneration on the Success of Surgical Endodontic Treatment of Teeth With Endodontic‐Periodontal Lesions: A Systematic Review,” International Endodontic Journal 56, no. 8 (2023): 910–921. 37212140 10.1111/iej.13936

